# Isolated Tubercular Liver Abscess in a Non-immunodeficient Patient: A Rare Case Report

**DOI:** 10.7759/cureus.6282

**Published:** 2019-12-03

**Authors:** Sujata Devi, Pritinanda Mishra, Madhusmita Sethy, Gargi Singh Thakur

**Affiliations:** 1 Internal Medicine, All India Institute of Medical Sciences, Bhubaneswar, IND; 2 Pathology, All India Institute of Medical Sciences, Bhubaneswar, IND

**Keywords:** tubercular liver abscess, liver abscess, hepatic tuberculosis

## Abstract

Isolated tubercular liver abscess (TLA) without the involvement of other organs is an extremely rare presentation of tuberculosis. This report describes a 23-year-old man who presented with a three-month history of fever and weight loss. Ultrasonography (USG) and contrast-enhanced computed tomography (CT) of the abdomen showed two abscesses in the liver, measuring 44 x 37 mm and 27 x 22 mm. Ultrasound-guided fine-needle aspiration was performed, with cytology confirming that the abscesses were tubercular. The patient was advised to start anti-tubercular therapy for six months.

Although rare, TLAs should be considered in the differential diagnosis of fine-needle aspiration of patients with liver abscesses and prolonged fever. Early diagnosis and timely intervention will prevent morbidity and mortality in such patients.

## Introduction

Liver abscess is an important cause of morbidity and mortality in tropical countries [1]. Worldwide, the etiology of liver abscess is either pyogenic or amoebic [2]. An isolated tubercular liver abscess (TLA) without the involvement of other organs is an uncommon presentation of extra-pulmonary tuberculosis and is usually associated with pulmonary and gastrointestinal tuberculosis in immunocompromised patients [2-3]. This report describes a non-immunodeficient patient diagnosed with TLA.

## Case presentation

**Figure 1:**
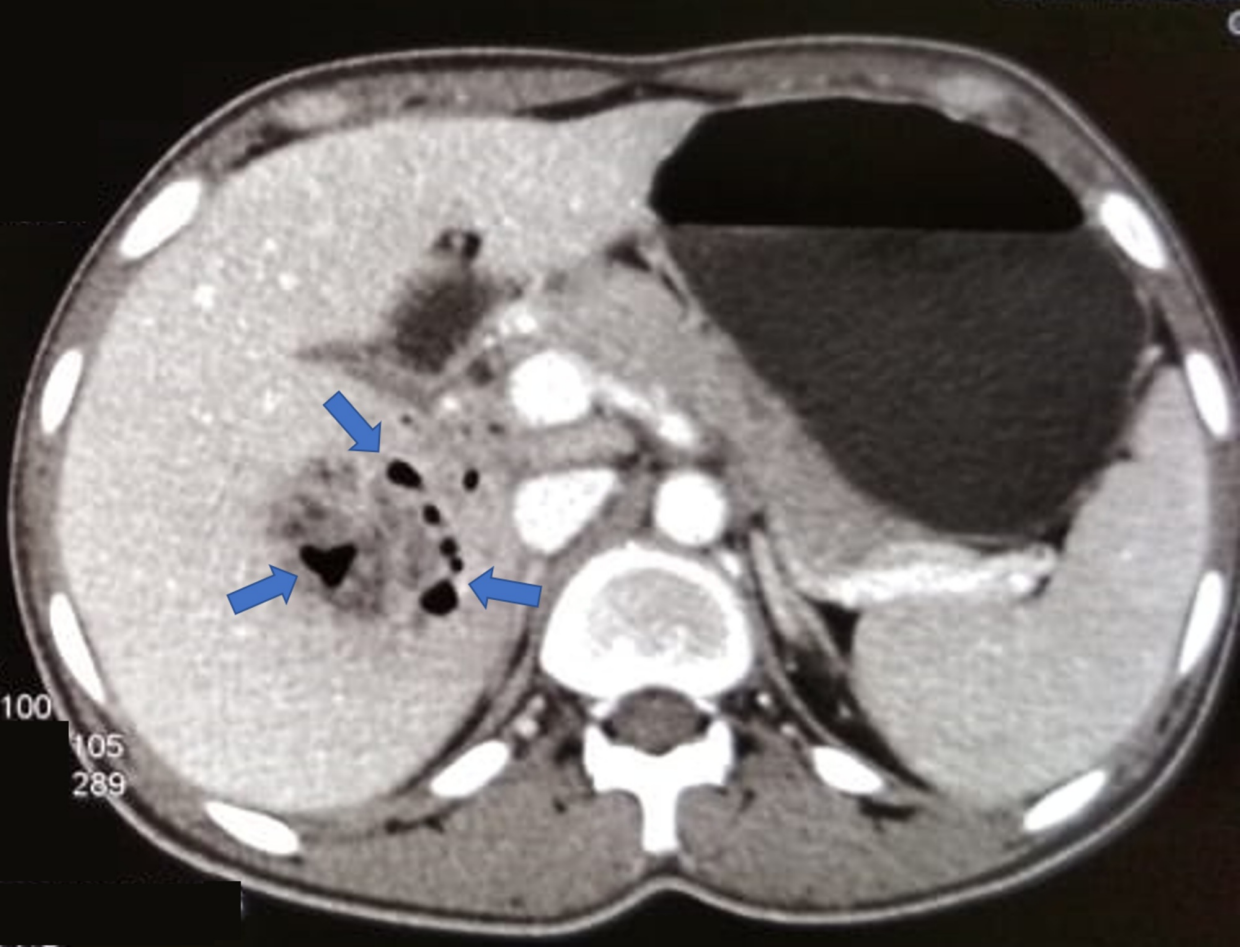
Contrast-enhanced computed tomography of the abdomen, showing liver abscesses

**Figure 2:**
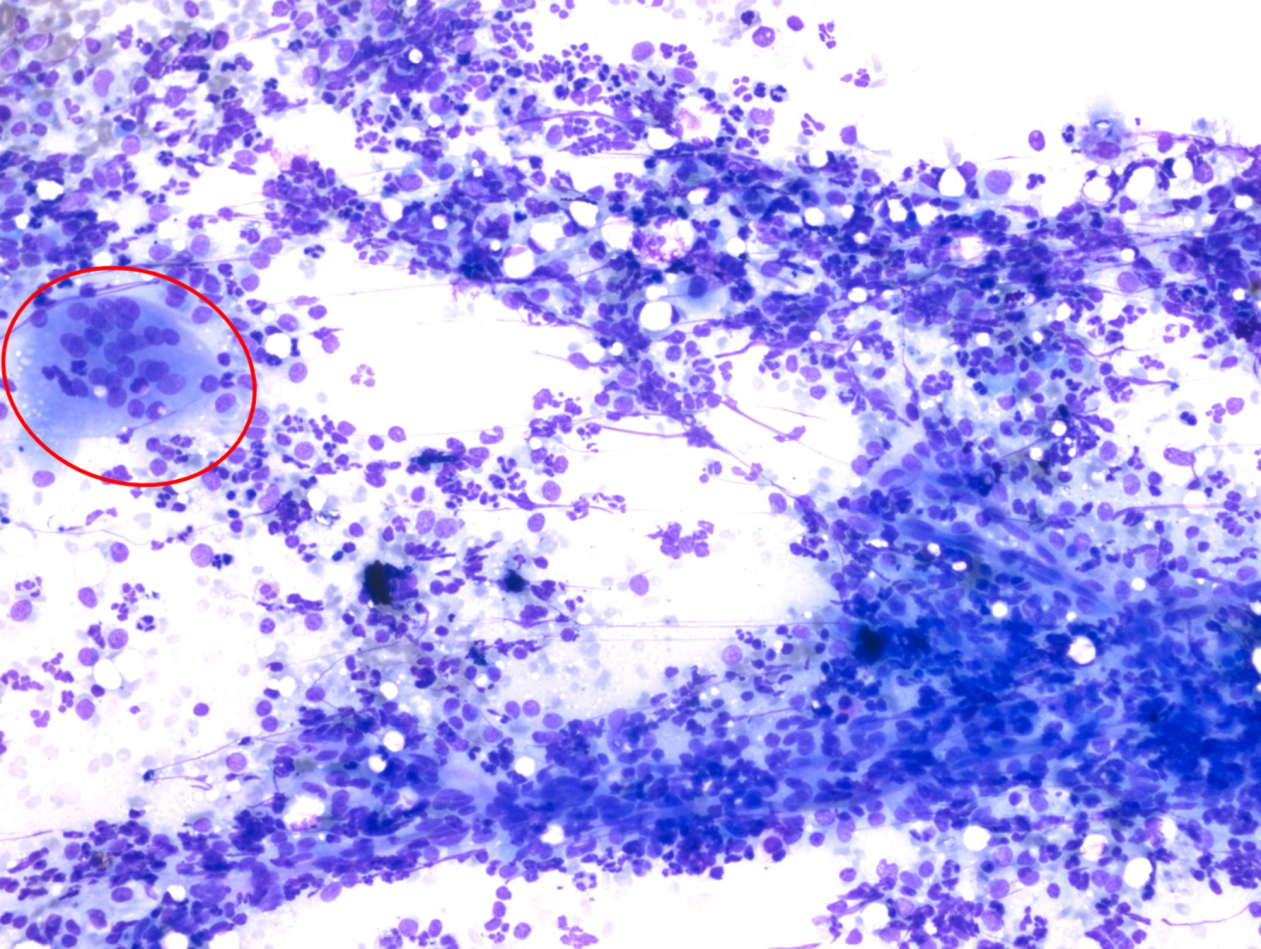
MGG staining of a liver biopsy sample, showing giant cell histiocytes on a neutrophilic background (20x magnification) MGG, May–Grünwald–Giemsa

**Figure 3:**
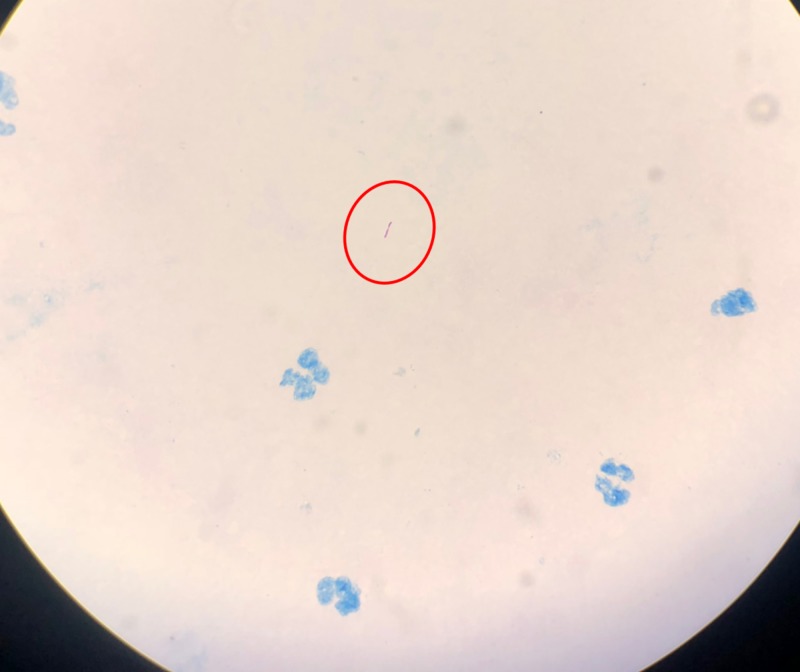
Ziehl–Neelsen staining of a liver biopsy sample, showing acid-fast Bacilli (with 100x magnification)

**Figure 4:**
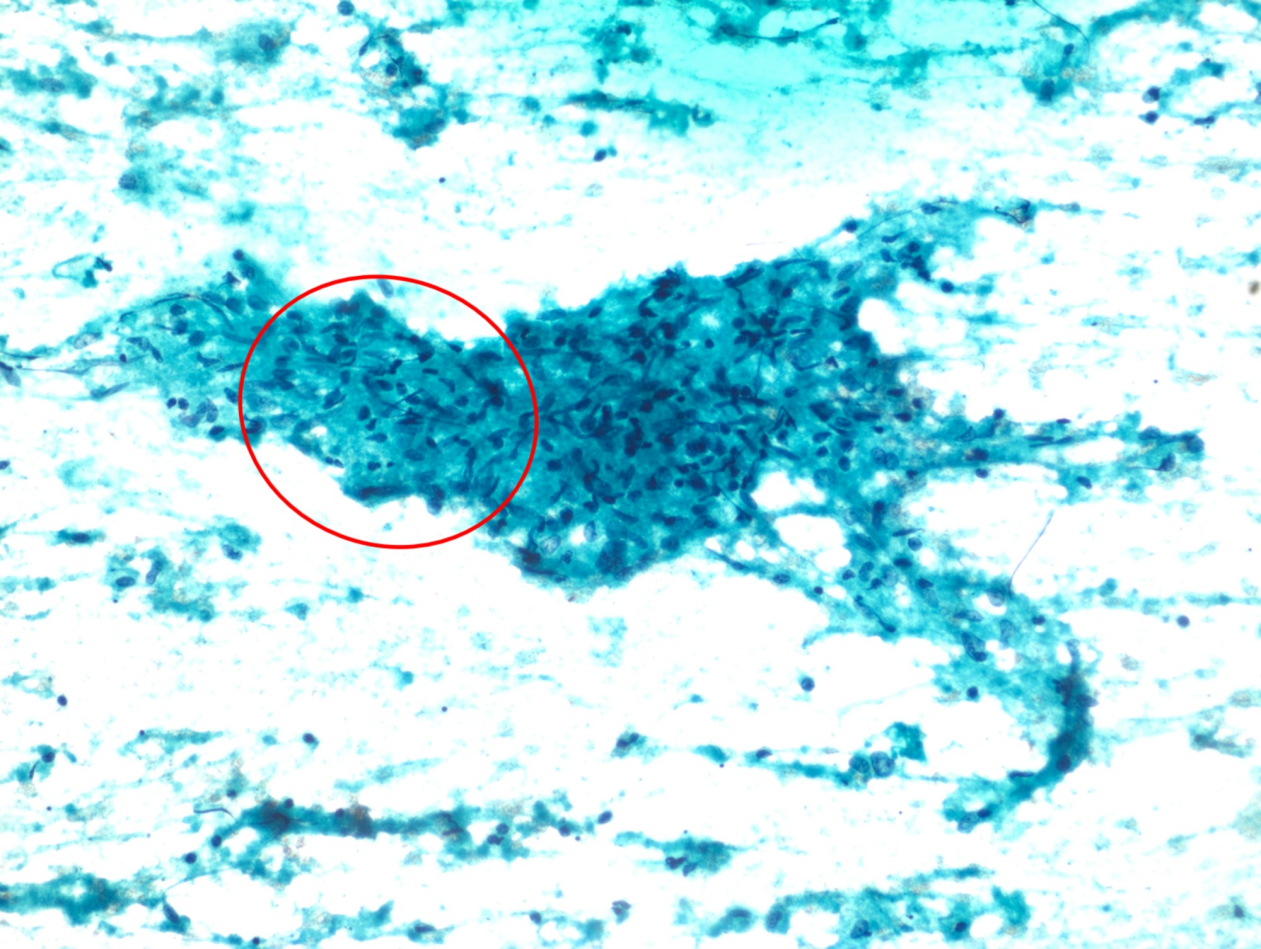
Papanicolaou staining of a liver biopsy sample, showing an epithelioid cell granuloma on a background of necrosis (10x magnification)

A 23-year-old man presented with fever and weight loss lasting three months. He had a history of occasional vomiting and pain in the abdomen, especially after intake of food. The patient had no history of tuberculosis or any contact with a person with tuberculosis. On examination, the patient was febrile, with a body temperature of 101 ºF (38.3 ºC). At admission, his pulse was 90 beats per minute, and his blood pressure was 106/80 mmHg. The patient was pale and weak, with no icterus or lymphadenopathy. The heart and lung physical examination was normal. Abdominal examination showed no evidence of tenderness or hepatosplenomegaly. The hemoglobin concentration was 9.6 g/dl, total leucocyte count was 13,000/mm^3^, and erythrocyte sedimentation rate was high, 106 during the first hour. Total platelet count was 8.66 x 106/mm^3^, serum bilirubin concentration was 0.9 mg/dl, and total serum protein concentration was 8.8 gm%, with albumin and globulin concentrations of 3.2 g% and 5.6 g%, respectively. Other liver function tests were within reference limits, and he was serologically negative for hepatitis B surface antigen, hepatitis C virus, and human immunodeficiency virus. The serum carbohydrate antigen (19-9) concentration was <2 U/ml, alpha-fetoprotein concentration was 1.94 IU/ml, and carcinoembryonic antigen concentration was 1.6 ng/ml. Non-contrast-enhanced computed tomography (CT) of the thorax revealed no significant abnormality. Ultrasonography (USG) and contrast-enhanced CT of the abdomen detected two abscesses in the liver measuring 44 × 37 mm and 27 x 22 mm (Figure 1). Ultrasound-guided fine-needle aspiration was performed, with cytology confirming the abscesses were tubercular (Figures 2-4). The patient was advised to start anti-tubercular therapy for six months.

## Discussion

The prevalence of isolated hepatic tuberculosis is around 0.34% [4]. Patients with hepatic tuberculosis present with fever, weight loss, and abdominal pain, as well as with other findings such as hepatosplenomegaly and pain in the right hypochondrium [3,5]. Laboratory testing shows leukocytosis and anemia, whereas liver function tests and bilirubin levels are usually within reference limits [6]. Hepatic tuberculosis is also characterized by a reversal in albumin and globulin concentrations, a finding observed in our patient. TLAs are frequently confused with pyogenic and amoebic liver abscess and with hepatoma [7]. USG and CT scans are diagnostic modalities with low specificity, with their findings reflecting different stages of the disease [8]. TLA is diagnosed by the detection of tubercular *Bacilli* in aspirated pus, by positive staining of liver biopsy for acid-fast Bacillus, or by culture of or polymerase chain reaction (PCR) assays for *Mycobacterium tuberculosis* [3]. PCR may be used for the rapid diagnosis of *M. tuberculosis* in clinical samples [9]. Treatment options include anti-tubercular therapy alone or combined with percutaneous aspiration [7]. 

## Conclusions

Although liver abscesses are common in tropical countries, isolated tubercular abscesses are rare, particularly in immunocompromised patients. If there is a history of fever with liver abscess, then one should have a suspicion about isolated tubercular abscesses. The diagnosis depends upon imaging studies such as USG and CT scan of the abdomen, whereas isolation of tubercular Bacilli in pus aspirate is more conclusive of diagnosis. Timely intervention will prevent morbidity and mortality in such patients. In our case, we started with anti-tubercular drugs, and the patient was doing well during follow-up.
